# Estrogens protect male mice from obesity complications and influence glucocorticoid metabolism

**DOI:** 10.1038/ijo.2015.102

**Published:** 2015-06-30

**Authors:** R S Dakin, B R Walker, J R Seckl, P W F Hadoke, A J Drake

**Affiliations:** 1 Endocrinology Unit, University/British Heart Foundation Centre for Cardiovascular Science, University of Edinburgh, The Queen's Medical Research Institute, Edinburgh, UK

## Abstract

**Background:**

Although the prevalence of obesity is higher among women than men, they are somewhat protected from the associated cardiometabolic consequences. The increase in cardiovascular disease risk seen after the menopause suggests a role for estrogens. There is also growing evidence for the importance of estrogen on body fat and metabolism in males. We hypothesized that that estrogen administration would ameliorate the adverse effects of obesity on metabolic parameters in males.

**Methods:**

Male and female C57Bl/6 mice were fed control or obesogenic (DIO) diets from 5 weeks of age until adulthood. Glucose tolerance testing was performed at 13 weeks of age. Mice were killed at 15 weeks of age and liver and adipose tissue were collected for analysis of gene expression. A second cohort of male mice underwent the same experimental design with the addition of estradiol pellet implantation or sham surgery at 6 weeks.

**Results:**

DIO males had greater mesenteric adipose deposition and more severe increases in plasma glucose, insulin and lipids than females. Treatment of males with estradiol from 6 weeks of age prevented DIO-induced increases in adipose tissue mass and alterations in glucose–insulin homeostasis. We also identified sex differences in the transcript levels and activity of hepatic and adipose glucocorticoid metabolizing enzymes. Estrogen treatment feminized the pattern of DIO-induced changes in glucocorticoid metabolism, rendering males similar to females.

**Conclusions:**

Thus, DIO induces sex-specific changes in glucose–insulin homeostasis, which are ameliorated in males treated with estrogen, highlighting the importance of sex steroids in metabolism. Given that altered peripheral glucocorticoid metabolism has been observed in rodent and human obesity, our results also suggest that sexually dimorphic expression and activity of glucocorticoid metabolizing enzymes may have a role in the differential metabolic responses to obesity in males and females.

## Introduction

There has been a rapid increase in the worldwide prevalence of obesity over recent decades, with a parallel increase in obesity-associated cardiometabolic disorders.^1^ Although the prevalence of obesity is higher among women, they may be somewhat protected from the associated cardiometabolic consequences, at least until the menopause.^2^ This is also the case in rodents: male mice are more prone to diet-induced obesity^3, 4^ and metabolic dysfunction^5, 6^ than females and there are sex differences in the response to a high-fat diet in genetic models of obesity and diabetes in rats.^7, 8^ A role for sex steroids, particularly estrogens, in modulating glucose and insulin homeostasis is supported by observations in humans: pre-menopausal women have higher insulin sensitivity when compared with age-matched men;^9, 10^ and following the menopause, women tend to accumulate visceral fat and become more insulin resistant, with a consequent increase in the risk of type 2 diabetes.^11^ An increasing body of evidence suggests that estrogens also have important beneficial effects on body fat and metabolism in males.^12, 13, 14, 15^ We used a mouse model of diet-induced obesity to investigate sex differences in susceptibility to the metabolic consequences of obesity by addressing the hypothesis that estrogen treatment in males would ameliorate the adverse effects of diet-induced obesity on metabolic parameters. Given that altered peripheral glucocorticoid metabolism has been observed in rodent and human obesity, we also sought to explore the potential role of sex differences in glucocorticoid metabolism.

## Materials and methods

### Animals and experimental design

All animal procedures were carried out under UK Home Office license approval under the Animals (Scientific Procedures) Act, 1986, and with local ethical committee approval. C57Bl/6 mice were bred in-house and maintained under controlled conditions of light (on 0700–1900 hours) and temperature (21 °C) with free access to food and water. Males and females (*n*=8 per group) were weaned aged 3 weeks onto standard chow (Special Diet Services Witham, Essex, UK) and at 5 weeks were randomly assigned by a technician not involved in the research to high-fat, high-sugar ‘obesogenic' diet (DIO: D12328, Research Diets, New Brunswick, NJ, USA) or control diet (CON: D12331, Research Diets). D12328 induces obesity in rodents^16^ and the corresponding control diet was matched in terms of protein and micronutrient content. Animals were weighed weekly and after 8 weeks were individually housed to allow for metabolic investigation. Animals were killed aged 15 weeks by CO_2_ asphyxiation, between 1400 and 1600 hours following a 6-h fast, and trunk blood was collected. Tissues were dissected immediately, weighed and snap frozen on dry ice. Females were killed during estrus, confirmed by vaginal smear examination showing exclusively non-nucleated cornified epithelial cells.

In a separate experiment, pre-pubertal C57Bl/6 mice of both sexes were killed following weaning (3–4 weeks of age; *n*=7 per group), after a physical examination to exclude puberty (vaginal opening in females and balanopreputial separation in males). Tissues were dissected immediately, weighed and snap frozen on dry ice.

A second cohort of male mice underwent the same experimental design with the addition of estradiol pellet implantation or sham surgery at 6 weeks (1 week post initiation of experimental diet; *n*=11 per sham group, 12 per estradiol group). Animals were anaesthetized using isoflurane and 90-day, 0.25 mg 17β-estradiol pellets (Innovative Research of America, Sarasota, FL, USA) implanted subcutaneously in the left mid-dorsal region. Skin incisions were closed using silk sutures. Sham-operated animals underwent the same surgical procedure with the exclusion of the pellet being inserted. Animals were killed aged 15 weeks using the same protocol as listed in experimental design. Plasma concentrations of 17β-estradiol were determined using an enzyme-linked immunosorbent assay (Calbiotech, Spring Valley, CA, USA) on blood samples taken when animals were killed.

### Metabolic measures

Glucose tolerance tests were performed at 13 weeks of age; animals were fasted for 6 h and tests commenced at 1400 hours. A basal blood sample was taken by tail nick followed by an intraperitoneal injection of a glucose load (2 g kg^−1^) and blood samples collected 15, 30, 60 and 90 min post injection. Samples were centrifuged at 2.3 *g* for 10 min and plasma stored at −20 °C. Plasma glucose, cholesterol and triglyceride concentrations were measured by enzymatic methods (ThermoElectron, Pittsburgh, PA, USA). Insulin (Crystal Chem Inc., Chicago, IL, USA) and testosterone (Demeditec Diagnostics GmbH, Kiel, Germany) were determined by enzyme-linked immunosorbent assay. Non-esterified fatty acid concentrations were determined using a kit (Wako Diagnostics, Richmond, VA, USA). At 14 weeks of age, plasma corticosterone was measured in tail tip blood samples taken at 0700 and 1900 within 1 min of cage disturbance, using an in-house radio-immuno assay.^17^ Hepatic triglyceride was extracted by saponification^18^ and quantified as in plasma.

### Quantification of mRNA

Total RNA was extracted from snap frozen tissues using an RNeasy mini kit (Qiagen, West Sussex, UK) and reverse transcribed (250 ng liver and 500 ng adipose) using the Access RT-PCR kit from Promega (Hampshire, UK). Real-time PCR was performed using a Roche Lightcycler 480 (Roche Diagnostics Ltd., Sussex, UK) with Universal Probes master mix (Roche) and thermal cycler parameters recommended by the manufacturer. PCR was performed in triplicate in a total volume of 10 μl containing 2 μl of complementary DNA, 5 μl Roche master mix, 12 pmol μl^−1^ primers and 4  pmol μl^−1^ probe (Universal probe library, Roche); primer sequences and probe numbers are given in Table 1. A standard curve was constructed for each primer/probe set using a serial dilution of complementary DNA pooled from all samples. Expression was corrected for cyclophilin A (Taqman gene expression assay Applied Biosytems, Warrington, UK; Mm02342430_g1) in the liver and beta-actin (Mm01215647_g1) in adipose tissue.

### 11β-HSD1 activity assay

11β-HSD1 is a reductase *in vivo*, converting inactive 11-dehydrocorticosterone to corticosterone. However, *in vitro,* dehydrogenase activity predominates and measurements of reductase activity are confounded by competition with other enzymes. Therefore, as an index of total 11β-HSD1 protein, we measured enzyme activity as conversion of corticosterone to 11-dehydrocorticosterone in the presence of an excess of the cofactor NADP^+^ as previously described.^19^ Reactions were optimized to ensure first-order kinetics (liver 0.025 mg ml^−1^ for 2–4 h; adipose 0.025–0.1 mg ml^−1^ for 16–24 h).

### Statistical analysis

Data presented are mean±s.e.m. Numbers were based on our previous studies.^20^ Statistical analysis was carried out by *t*-testing, two-way or repeated measures analysis of variance as appropriate. Data were normally distributed and groups compared had similar variances. Values were considered different when *P*<0.05.

## Results

### Effects of DIO on glucose homeostasis, lipids and plasma corticosterone in males and females

**Figure 1:**
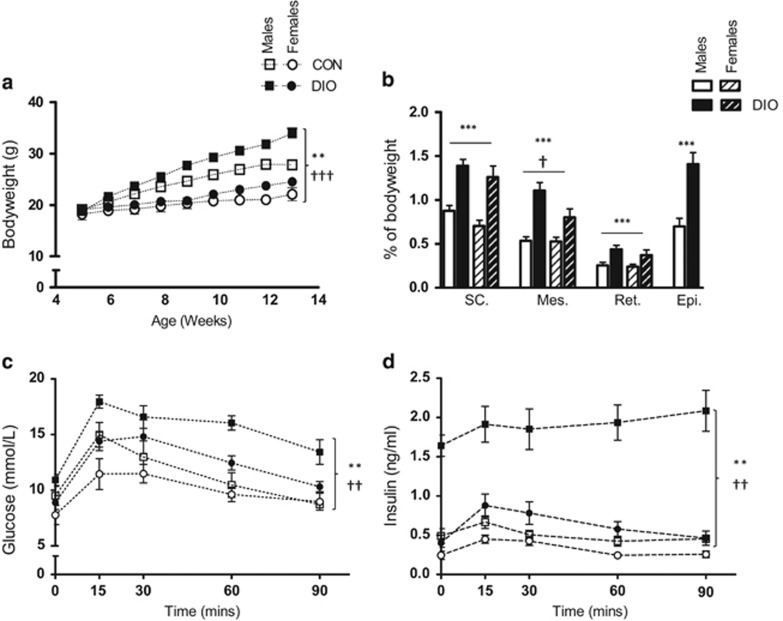
Effect of obesogenic diet and sex on adiposity and metabolism in C57Bl/6 mice. Male and female mice were fed control (CON) or obesogenic diet (DIO) from 5 weeks of age. (**a**) Body weight during the study, (**b**) adipose mass as percentage of body weight at killing. SC, subcutaneous; Mes, mesenteric; RP, retroperitoneal; Epi, epididymal. Plasma glucose (**c**) and insulin (**d**) during a glucose tolerance test. Area under curve (AUC) glucose: male CON 1003.2±97.5; male DIO 1285.2±93.7; female CON 859.1±65.8; female DIO 961.5±126.3. AUC insulin: male CON 38.9±6.9; male DIO 160.0±22.3; female CON 28.9±1.9; female DIO 55.7±7.4. Data are mean±s.e.m., *n*=8 per group, analyzed by repeated measures analysis of variance (ANOVA) (**a**, **c** and **d**) and two-way ANOVA (**b**), repeated measures where necessary. Effect of diet **P<0.01, ***P<0.001; effect of sex ^†^*P*<0.05, ^††^*P*<0.01, ^†††^*P*<0.001.

Consumption of an obesogenic diet caused a significant increase in body weight in males (Figure 1a) and an increase in adipose tissue weights in both the sexes (Figure 1b). Plasma triglyceride concentrations were lower in CON females compared with males (Table 2) but plasma cholesterol and plasma and hepatic triglyceride concentrations were similarly increased by DIO in both the sexes (Table 2). DIO increased plasma concentrations of glucose (Figure 1c) and insulin (Figure 1d) following a glucose load, with a substantially larger effect in males. In mice on control diet, nadir plasma corticosterone concentrations were comparable in both the sexes; however, peak concentrations were higher in females than in males (Table 2). Peak plasma corticosterone was reduced by DIO in both the sexes (Table 2).

### Effects of estradiol administration in male mice

**Figure 2:**
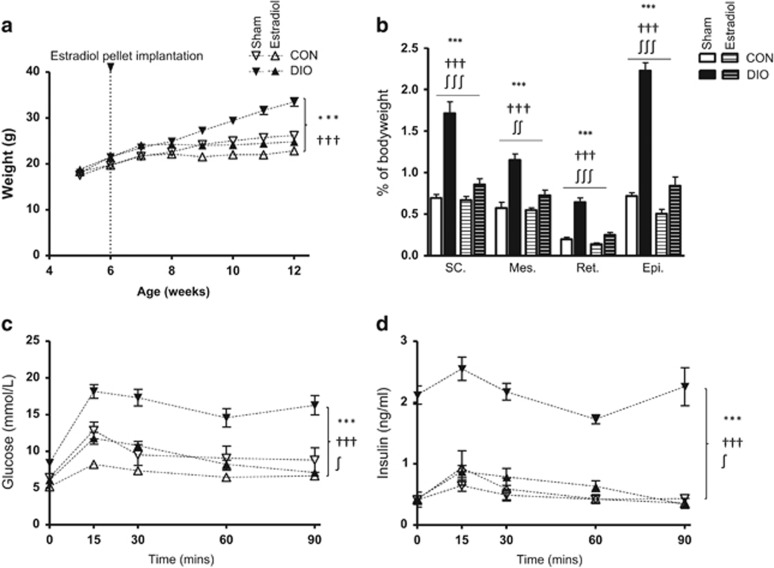
Effect of obesogenic diet and estrogen treatment on adiposity and metabolism in male mice. Male mice were fed control (CON) or obesogenic (DIO) diet from 5 weeks of age and at 6 weeks underwent implantation of a continuous release 17β-estradiol pellet (estradiol) or sham surgery (sham). (**a**) Body weight over duration of study. (**b**) Adipose mass as percentage of body weight. SC, subcutaneous; Mes, mesenteric; RP, retroperitoneal; Epi, epididymal. Plasma glucose (**c**) and insulin (**d**) during a glucose tolerance test. AUC glucose: sham CON 859.2±123.7; sham DIO 1290.6±110.8; estrodiol CON 596.9±32.3; estrodiol DIO 759.3±46.3. AUC insulin: sham CON 41.0±5.8; sham DIO 185.8±9.9; estrodiol CON 44.8±10.7; estrodiol DIO 55.0±6.5. Data are mean±s.e.m., *n*=11 per sham group, 12 per estradiol group, analyzed by repeated measures analysis of variance (ANOVA) (**a**, **c** and **d**) or two-way ANOVA (**b**), repeated measures where necessary. Effect of diet ***P<0.001; effect of estradiol ^†††^*P*<0.001; interaction between effects ^∫^*P*<0.05, ^∫∫^*P*<0.01, ^∫∫∫^*P*<0.001.

At the end of the experiment, plasma estradiol concentrations were increased in males treated with estradiol compared with the sham group, and were significantly higher in the estradiol-treated DIO group compared with all other groups (Table 3). Plasma testosterone concentrations were reduced by estradiol treatment but unaffected by diet (Table 3). Estradiol administration reduced weight gain (Figure 2a) and adipose tissue weight (Figure 2b) in both CON and DIO males. Estradiol also ameliorated the increase in plasma glucose and insulin caused by DIO (Figures 2c and d) but did not affect plasma triglyceride or cholesterol concentrations (Table 3). However, estrogen treatment reversed the DIO-associated increase in hepatic triglyceride (Table 3). Nadir plasma concentrations of corticosterone were higher in estradiol-treated males but were unaffected by DIO; peak concentrations were comparable in all the groups (Table 3).

### Glucocorticoid metabolism in DIO and the effects of estrogen

**Figure 3:**
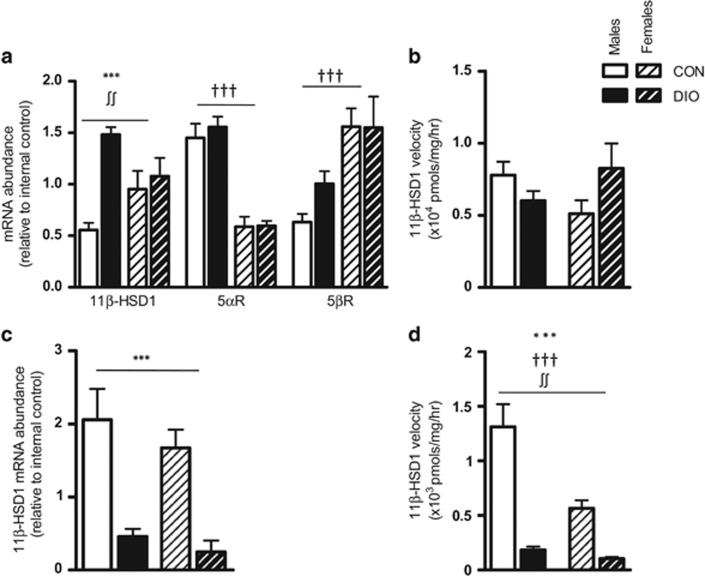
Effect of obesogenic diet and sex on glucocorticoid metabolism. Male and female mice were fed control (CON) or obesogenic diet (DIO) from 5 weeks of age. mRNA abundance of glucocorticoid metabolizing genes was assessed using real-time PCR in the liver (**a**) and subcutaneous adipose (**c**). Activity of 11β-HSD1 was analyzed in samples from the same tissues (**b** and **d**). Data are mean±s.e.m., *n*=8 per group, analyzed by two-way analysis of variance. Effect of diet ***P<0.001; effect of sex ^†††^*P*<0.001; interaction between diet and sex ^∫∫^*P*<0.01.

Given the sex differences in circulating corticosterone levels and the fact that altered glucocorticoid metabolism may contribute to the pathogenesis of obesity and related metabolic disorders,^21^ we proceeded to measure transcript and expression levels of glucocorticoid metabolizing enzymes including 11β-hydroxysteroid dehydrogenase type 1 (11β-HSD1, which reactivates glucocorticoids from their inert 11-keto metabolites) and the levels of the A-ring reductases (5α- and 5β-reductase, which convert glucocorticoids into their dihydro metabolites). Females had lower hepatic transcript levels of 5α-reductase and higher transcript levels of 5β-reductase than males (Figure 3a). There was a trend for females to have higher hepatic transcript levels of *11β-HSD1* than males (*P*=0.057; Figure 3a) but there were no differences in hepatic enzyme activity (Figure 3b). In subcutaneous fat, although there were no sex differences in transcript levels of *11β-HSD1* (Figure 3c), enzyme activity was lower in females compared with males (Figure 3d). DIO increased hepatic transcript levels of *11β-HSD1* in males but not in females (Figure 3a), although there were no changes in hepatic enzyme activity (Figure 3b). There was no effect of DIO on hepatic transcript levels of 5α-reductase or 5β-reductase in either sex (Figure 3a). In subcutaneous adipose tissue, DIO reduced 11β-HSD1 expression (Figure 3c) and activity (Figure 3d) in both the sexes.

**Figure 4:**
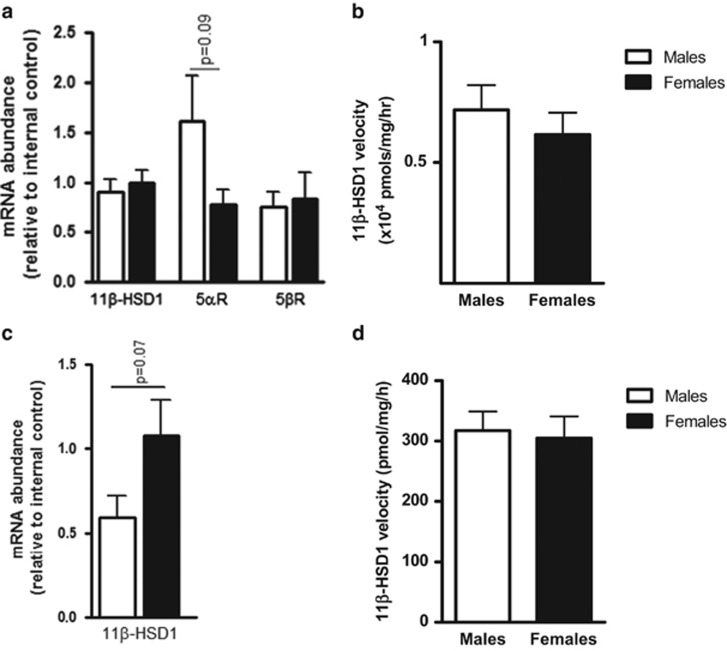
Sex differences in hepatic and subcutaneous fat glucocorticoid metabolism in pre-pubertal mice. Male and female mice were killed aged 3–4 weeks, before signs of puberty, and liver and subcutaneous adipose were collected. mRNA abundance of glucocorticoid metabolizing genes was assessed using real-time PCR in liver (**a**) and subcutaneous fat (**c**) and 11β-HSD1 activity determined in liver (**b**) and subcutaneous fat (**d**). Data are mean±s.e.m., *n*=7 per group, analyzed by Student's *t*-test.

To investigate when the sex differences in glucocorticoid metabolizing enzymes became apparent, a cohort of mice were killed before puberty (when sex-steroid concentrations are low) and enzyme expression and activity were determined. In pre-pubertal mice, there was a trend for a lower expression of hepatic 5α-reductase in females (Figure 4a), but no differences in the expression of hepatic 5β-reductase or in the expression or activity of 11β-HSD1 (Figures 4a and b). Furthermore, expression and activity of 11β-HSD1 in adipose tissue were comparable in male and female pre-pubertal mice (Figures 4c and d).

**Figure 5:**
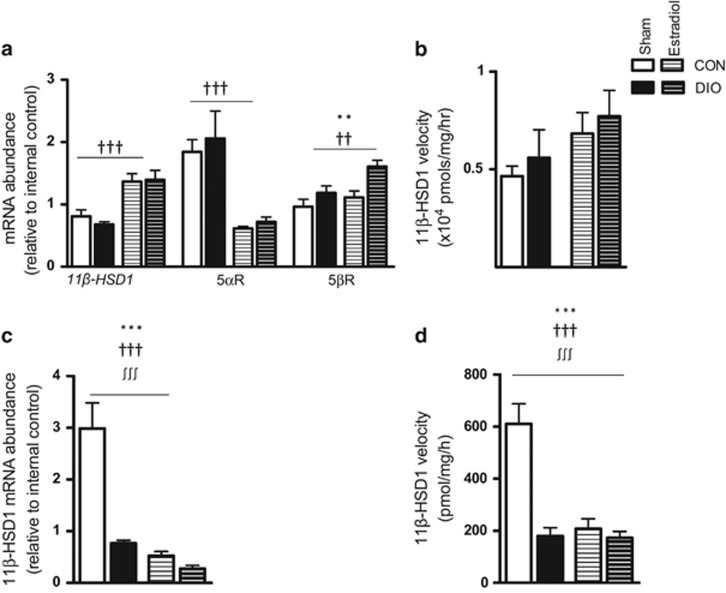
Effect of obesogenic diet and estrogen treatment on glucocorticoid metabolism in male mice. Male mice were fed control (CON) or obesogenic (DIO) diet from 5 weeks of age and at 6 weeks underwent implantation of a continuous release 17β-estradiol pellet (estradiol) or sham surgery (sham). mRNA abundance was assessed using real-time PCR in the liver (**a**) and subcutaneous adipose (**c**). Activity of 11β-HSD1 was analyzed in samples from the same tissues (**b** and **d**). Data are mean±s.e.m., *n*=11 per sham group, 12 per estradiol group, analyzed by two-way analysis of variance. Effect of diet **P<0.01, ***P<0.001; effect of estradiol ^††^*P*<0.01, ^†††^*P*<0.001; interaction between effects ^∫∫∫^*P*<0.001.

In adult males, estradiol increased hepatic transcript levels, but not enzyme activity, of 11β-HSD1 (Figures 5a and b). Analysis by two-way analysis of variance revealed an effect of estradiol to reduce hepatic transcript levels of 5α-reductase and increase transcript levels of 5β-reductase in both CON and DIO males (Figure 5a). In adipose tissue, by contrast with the liver, analysis by two-way analysis of variance revealed an overall effect of estradiol to reduce both the expression (Figure 5c) and activity (Figure 5d) of 11β-HSD1 in CON and DIO males. As DIO reduces both expression and activity of 11β-HSD1 in adipose, resulting in lower baseline 11β-HSD1 levels, there was a much smaller relative reduction in both expression and activity in the estradiol-treated DIO group (Figures 5c and d).

## Discussion

Our data confirm previous reports showing that male mice are markedly more susceptible than females to the effects of an obesogenic diet.^3, 4, 5, 6^ In postmenopausal women and in female animal models, lower estrogen levels are associated with increased visceral adiposity^22, 23^ and estrogen replacement improves glucose–insulin homeostasis.^24, 25^ We show that estrogen treatment of males is associated with an improvement in weight gain and DIO-induced metabolic changes, supporting the concept that estrogen has an important role in the control of metabolism and adiposity. There is growing evidence for a fundamental role for estrogen in the regulation of obesity and related metabolic disorders in males^12, 13, 14^ and recent data from rodent studies suggest that hepatic estrogen signaling has a key role in the prevention of high-fat diet-induced insulin resistance in males.^26^ Indeed, the aromatization of testosterone to estradiol may underpin many of the physiological effects which have generally been attributed to the action of testosterone, for example, the prevention of visceral adiposity.^12^ Estrogen may also have a role in the control of hepatic lipid metabolism and hepatic lipid deposition,^26, 27, 28^ and estrogen treatment in DIO males abolished the DIO-induced increase in the accumulation of hepatic triglyceride. Intriguingly, this occurred in the absence of any effect of estradiol on plasma triglyceride and cholesterol concentrations. Estrogen may influence appetite and energy expenditure.^29^ Although we did not measure food intake and energy expenditure in this study, data from female rodents suggest that ovariectomy induces an increase in food intake and estrogen replacement decreases food intake.^29^ However, hyperphagia does not fully account for the changes in metabolism and development of obesity after ovariectomy.^29^ Our findings, showing the importance of estrogen signaling in regulating body weight, glucose–insulin homeostasis and hepatic triglyceride content are in agreement with studies in mice lacking the estrogen receptor (ER). Mice lacking ER have increased adipose tissue, higher fasting blood glucose and insulin^30^ and hepatic insulin resistance with altered hepatic lipid handling.^26, 31^ Furthermore, deletion of ERα in mice blocks the antiobesity effects of estrogen replacement.^29^ Male mice lacking ER specifically in the liver show reduced insulin sensitivity.^26^ Conversely, hepatic ERα overexpression is associated with markedly reduced hepatic triglyceride content and improved insulin sensitivity.^32^

There were sex differences in circulating corticosterone concentrations and, as in previous rodent studies, DIO reduced peak corticosterone concentrations in both the sexes,^33, 34^ which may reflect altered peripheral glucocorticoid clearance.^35, 36^ As altered glucocorticoid metabolism may contribute to the pathogenesis of obesity and related metabolic disorders,^21^ we hypothesized that some of the protective effects of estrogen might be due to effects on adipose and/or hepatic glucocorticoid metabolism. There are sex differences in the expression and activity of glucocorticoid metabolizing enzymes in humans, with lower expression and activity of hepatic and adipose 11β-HSD1 in females^37^ and estrogen regulates 11β-HSD1 expression and activity in the rat liver^38^ and kidney,^39^ and in rodent and human adipocytes.^40, 41^ We observed sex-specific responses of glucocorticoid metabolizing enzymes to diet-induced obesity, and estrogen therapy feminized the pattern of enzyme expression and activity in males. We observed no sex differences in the expression or activity of hepatic or adipose 11β-HSD1 in pre-pubertal mice, although there were clear sex differences in adult animals. 11β-HSD1 messenger RNA (mRNA) and activity were higher in lean males compared with females, a finding which has also been reported in humans.^37^ This predicts greater regeneration of corticosterone in adult male adipose tissue and may be a disadvantage given that mice overexpressing 11β-HSD1 in adipose tissue exhibit intra-abdominal obesity and metabolic dysfunction.^42^ The DIO-induced reduction in 11β-HSD1 mRNA and activity in subcutaneous adipose tissue that occurred in both the sexes in our study has previously been reported in male rodents^34, 43^ and has been proposed as a protective mechanism to reduce both circulating glucocorticoid concentrations and local glucocorticoid signaling.^34, 43^ Indeed, 11β-HSD1-knockout mice, or mice treated with selective 11β-HSD1 inhibitors, are resistant to obesity and hyperglycemia when fed a high-fat diet,^44, 45, 46, 47, 48^ although, notably, these studies were carried out in male animals. Lower adipose 11β-HSD1 in females compared with males may, therefore, contribute to the relative protection of females from the metabolic effects of obesity. Consistent with this hypothesis, the estrogen-induced reduction in adipose 11β-HSD1 mRNA and activity may be one mechanism for the protection from the metabolic consequences of high-fat diet in estrogen-treated males. However, the relative importance of changes in adipose tissue 11β-HSD1 in mediating the metabolic response to DIO remains unclear, as DIO itself is associated with a profound reduction in the expression and activity of adipose 11β-HSD1 in both the sexes, yet the metabolic phenotype is more severe in DIO males.

Sex differences in the expression of 5α- and 5β-reductase also became apparent after puberty. As 5α-reduced glucocorticoid metabolites are active at the glucocorticoid receptor,^49^ the increased expression of 5α-reductase and lower expression of 5β-reductase in males predict increased hepatic concentrations of active glucocorticoids in males and may render them more susceptible to the effects of a high-fat diet. Indeed, in humans obesity is associated with alterations in the ratio of urinary 5α- and 5β-reduced glucocorticoid metabolites, with an increased proportion of cortisol metabolized by 5α-reduction.^21, 36, 50^ Estradiol treatment in males decreased hepatic 5α-reductase and increased 5β-reductase expression, resulting in a similar expression pattern to that in females. As 5β-reductase can metabolize both corticosterone and 11-dehydrocorticosterone, and its metabolites are not active at glucocorticoid receptor, this predicts reduced intrahepatic glucocorticoid signaling and may be one mechanism by which estrogen treatment resulted in protection from the metabolic consequences of exposure to a high-fat diet. Although females, DIO males and estradiol-treated males had increased hepatic 11β-HSD1 expression in comparison to lean males, this was not reflected in differences in enzyme activity. We have previously suggested that this discrepancy may reflect post-transcriptional modification.^34^ Intriguingly, the DIO-induced increase in hepatic 11β-HSD1 and the reduction in peak corticosterone were not present in the sham surgery DIO males suggesting that there may be long-term effects of surgery on glucocorticoid metabolism.

In terms of mechanisms, changes in 11β-HSD1 expression following ovariectomy and estrogen treatment in female rats could be due to reduced adiposity rather than increases in estradiol.^51^ However, both direct and indirect effects of estradiol on adipose 11β-HSD1 have been proposed: estradiol is a competitive inhibitor of 11β-HSD1 in primary cultures of rat adipocytes^40^ and higher adipose expression of 11β-HSD1 is found in postmenopausal women.^41^ In our study, the lack of sex difference in adipose and liver 11β-HSD1 expression and activity in pre-pubertal mice, when sex-steroid concentrations are low, combined with the changes with estrogen treatment in adult males are consistent with direct regulation of glucocorticoid metabolism in adipose tissue. Although estrogen may have an important influence on peripheral glucocorticoid metabolism, there is increasing evidence for the key role of insulin.^52, 53, 54, 55^ In rodents, insulin senitization ameliorates the obesity-induced changes in hepatic A-ring reductase expression and activity,^55^ and in humans, intravenous insulin acutely increases cortisol regeneration by 11β-HSD1^(ref. 54)^ and cortisol production by 11β-HSD1 parallels the change in circulating insulin concentrations following meals.^52^ Thus, the changes in hepatic and adipose glucocorticoid metabolism seen in females and in estrogen-treated males may additionally represent a downstream effect of the marked improvement in insulin sensitivity. Finally, the changes induced by estrogen treatment may also be attributed to reduced testosterone levels in both CON and DIO males. However, a recent study in humans in which endogenous testosterone and estradiol were suppressed pharmacologically suggests that, whereas androgen deficiency may account for decreases in lean mass, estrogen deficiency is responsible for increases in body fat.^12^ Nevertheless, altered estrogen/androgen balance may still be of importance in the maintenance of normal physiology and future studies in which testosterone levels are maintained at physiological levels would help to determine the relative importance of estrogens versus androgens.

Our hypothesis for this study was that estrogen treatment in males would ameliorate the adverse effects of diet-induced obesity on metabolic parameters. Consequently, we did not assess the effects of administration of estradiol in females. Estrogens may also have a role in mediating glucose–insulin homeostasis in women and estrogen deficiency is associated with an increasing risk of obesity, the metabolic syndrome and type 2 diabetes.^29^ In postmenopausal women, the administration of estrogen can improve glucose homeostasis, insulin sensitivity and lipid profile.^56, 57, 58^ Studies using estrogen replacement in ovariectomized mice have shown that estrogen protects against fatty liver and may improve pathway-selective insulin resistance.^28^

In conclusion, our data support the importance of estrogen in the apparent protection of females from the deleterious effects of exposure to an obesogenic diet and provide further evidence for the suggestion that manipulating estrogen signaling pathways may represent an alternative/additional approach to the management of the complications of obesity in males. In addition, they suggest that sexually dimorphic expression and activity of glucocorticoid metabolizing enzymes may contribute to gender differences in metabolic responses to diet-induced obesity. Understanding the molecular basis of sex differences in disease risk may provide new approaches to the management of obesity-associated metabolic disease.

## Figures and Tables

**Table 1:** Primer sequences for quantitative real-time PCR

*Gene accession number*	*Primer sequence (5′-3′)*	*Probe number*
5α reductase (*5αR)* (NM_175283.3)	For—gggaaactggatacaaaataccc Rev—ccacgagctccccaaaata	41
5β-reductase (*5βR)* (NM_145364.2)	For—gaaaagatagcagaagggaaggt Rev—gggacatgctctgtattccataa	103
11β hydroxysteroid dehydrogenase type 1 (*11β-HSD1)* (NM_008288.2)	For—tctacaaatgaagagttcagaccag Rev—gccccagtgacaatcactt	1

**Table 2:** Concentrations of lipids and corticosterone in obese and control mice

	*Males*		*Females*		*Effect of diet*P-*value*	*Effect of sex*P*-value*	*Interaction diet and sex*P-*value*
	*CON*	*DIO*	*CON*	*DIO*			
Fasting plasma triglyceride (mmol l^−1^)	0.57±0.03	0.78±0.05	0.41±0.03	0.54±0.02	0.000027	0.000005	0.21
Total plasma cholesterol (mmol l^−1^)	1.71±0.13	2.67±0.23	1.53±0.17	2.86±0.16	<0.000001	0.55	0.56
Hepatic triglyceride (nmol mg^−1^)	27.5±3.7	60.6±8.6	26.5±3.0	58.5±3.8	0.000001	0.77	0.93
Nadir plasma corticosterone (nm)	64.8±8.0	70.8±7.6	66.6±14.3	62.1±10.0	0.94	0.74	0.60
Peak plasma corticosterone (nm)	282.0±43.0	177.0±26.2	457.3±62.0	375.0±33.1	0.03	0.0001	0.79

Male and female mice were fed control (CON) or obesogenic (DIO) diets from 5 weeks of age. Plasma lipid and corticosterone levels were measured in samples taken during metabolic tests. Hepatic triglyceride was analyzed in post-mortem tissue. Data are mean±s.e.m., analyzed by two-way analysis of variance *n*=8 per group.

**Table 3:** Concentrations of lipids, corticosterone and testosterone in male obese and control mice treated with estradiol

	*Sham*		*Estradiol*		*Effect of diet*P*-value*	*Effect of estradiol*P*-value*	*Interaction of effects*P*-value*
	*CON*	*DIO*	*CON*	*DIO*			
Fasting plasma triglyceride (mmol l^−1^)	0.55±0.05	0.94±0.09	0.59±0.06	0.81±0.06	0.000024	0.49	0.20
Total plasma cholesterol (mmol l^−1^)	2.19±0.31	3.59±0.44	2.05±0.22	3.16±0.21	0.00016	0.34	0.62
Hepatic triglyceride (nmol mg^−1^)	19.0±2.7	48.8±6.9	25.9±2.8	27.9±3.3	0.0005	0.10	0.0019
Nadir plasma corticosterone (nm)	15.3±3.5	17.0±2.9	50.0±11.7	39.6±6.0	0.55	0.00031	0.41
Peak plasma corticosterone (nm)	148.7±18.1	178.9±24.5	240.2±35.5	173.1±30.6	0.52	0.14	0.09
Plasma estradiol (pg ml^−1^)	2.5±0.4	2.0±0.3	6.9±1.2	21.6±4.7	0.09	<0.000001	0.011
Plasma testosterone (ng ml^−1^)	2.5±1.3	2.2±1.4	0.4±0.1	0.5±0.2	0.703	0.021	0.95

Male mice were fed control (CON) or obesogenic (DIO) diets from 5 weeks of age and at 6 weeks of age underwent implantation of a continuous release 17β-estradiol pellet (estradiol) or sham surgery (sham). Plasma lipid and corticosterone levels were measured in samples taken during metabolic tests. Hepatic triglyceride was analyzed in post-mortem tissue. Data are mean±s.e.m., analyzed by two-way analysis of variance, plasma estradiol concentrations were log transformed before analysis. *n*=11 per sham group, 12 per estradiol group.
